# Tyrosine Kinase Inhibitors Display Potent Activity against Cryptosporidium parvum

**DOI:** 10.1128/spectrum.03874-22

**Published:** 2022-12-19

**Authors:** Maria G. Nava, Félix Calderón, Elena Fernández, Lluis Ballell, Paul Bamborough, Sumiti Vinayak

**Affiliations:** a Department of Pathobiology, College of Veterinary Medicine, University of Illinois at Urbana-Champaign, Urbana, Illinois, USA; b Global Health, GlaxoSmithKline, Tres Cantos, Madrid, Spain; c Molecular Design, GlaxoSmithKline, Stevenage, Herts, United Kingdom; Weill Cornell Medicine

**Keywords:** *Cryptosporidium*, anti-parasitic compounds, cryptosporidiosis, kinase library screening, protozoan parasite, tyrosine kinase inhibitors

## Abstract

The protozoan parasite *Cryptosporidium* is a leading cause of diarrheal disease (cryptosporidiosis) and death in young children. Cryptosporidiosis can be life-threatening in individuals with weak immunity such as HIV/AIDS patients and organ transplant recipients. There is currently no effective drug to treat cryptosporidiosis in the pediatric and immunocompromised population. Therefore, there is an urgent need to expedite the drug discovery process in order to develop new and effective therapies to reduce the global disease burden of cryptosporidiosis. In this study, we employed a drug repurposing strategy to screen a library of 473 human kinase inhibitors to determine their activity against Cryptosporidium parvum. We have identified 67 new anti-cryptosporidial compounds using phenotypic screening based on a transgenic C. parvum strain expressing a luciferase reporter. Further, dose-response assays led to the identification of 11 hit compounds that showed potent inhibition of C. parvum at nanomolar concentration. Kinome profiling of these 11 prioritized hits identified compounds that displayed selectivity in targeting specific families of kinases, particularly tyrosine kinases. Overall, this study identified tyrosine kinase inhibitors that hold potential for future development as new drug candidates against cryptosporidiosis.

**IMPORTANCE** The intestinal parasite Cryptosporidium parvum is a major cause of diarrhea-associated morbidity and mortality in children, immunocompromised people, and young ruminant animals. With no effective drug available to treat cryptosporidiosis in humans and animals, there is an urgent need to identify anti-parasitic compounds and new targets for drug development. To address this unmet need, we screened a GSK library of kinase inhibitors and identified several potent compounds, including tyrosine kinase inhibitors, that were highly effective in killing C. parvum. Overall, our study revealed several novel compounds and a new family of kinases that can be targeted for anti-cryptosporidial drug development.

## INTRODUCTION

*Cryptosporidium* spp. (C. parvum and C. hominis) are recognized as one of the leading causes of diarrhea-associated morbidity and mortality in children under 2 years of age living in resource-poor settings (1–4). Cryptosporidiosis is also a major cause of severe malnutrition resulting in growth stunting and developmental defects in children under 5 years of age that encounter multiple diarrheal episodes (2, 5). In addition to causing pediatric diarrhea, *Cryptosporidium* is an opportunistic pathogen that is a common cause of infection in immunocompromised individuals such as HIV/AIDS patients and organ transplant recipients (6, 7). While C. hominis is anthroponotic and only infects humans, C. parvum is a zoonotic pathogen and thus can infect both animals and humans. In fact, C. parvum is a common cause of neonatal diarrhea (“scours”) and mortality in ruminant livestock particularly calves (8). Transmission of infection to humans occurs by uptake of water or food contaminated with *Cryptosporidium* oocysts or via contact with infected animals. Cryptosporidiosis is not only limited to resource-poor countries but is a major cause of frequent outbreaks associated with recreational water use in the United States and other developed countries (9). There is no drug to effectively treat cryptosporidiosis and no vaccine available to prevent infection in humans and ruminants. The single US FDA approved drug for human use, nitazoxanide, has poor efficacy in malnourished children and immunocompromised individuals and is not commonly used in clinical settings for treatment (10–12). Thus, there is an urgent need for the development of new anti-cryptosporidal therapies that are safe and effective for the pediatric and immunocompromised groups, in order to reduce the global disease burden.

Over the past few years, there have been rapid advancements in the development and application of *in vitro* and *in vivo* assays to accelerate the discovery of new compounds against *Cryptosporidium* (13–19). Both phenotypic screening and target-based drug discovery approaches have been employed to identify compounds that inhibit *Cryptosporidium* growth *in vitro*, and lead candidates have been shown to be efficacious in immunocompromised mouse models of infection (10, 14, 15, 20). Some of the leading anti-cryptosporidial compounds identified from these approaches are inhibitors of parasite kinases and aminoacyl tRNA synthetases (14, 20–22). Two preclinical candidates, KDU731 and bumped kinase inhibitor BKI-1369, that target the parasite’s lipid kinase PI(4)K and calcium-dependent protein kinase 1 (CDPK1), respectively, have also been shown to be effective in reducing oocyst shedding and diarrheal illness in the calf clinical model of cryptosporidiosis (14, 23).

Strategies that have been successfully applied to expedite the drug discovery process against parasitic diseases include drug repurposing or target repositioning (20, 24–28). Drug repurposing involves the identification of new uses of approved drugs, while target repurposing, for infectious diseases, involves the matching of pathogen targets with their human orthologs in which hits/leads have been described (29, 30). Among infectious diseases, other pragmatic approaches to accelerate new drug-discovery efforts include the repurposing of bioactive compounds with activity in similar pathogens (26). High-throughput screening of kinase libraries such as the publicly available GlaxoSmithKline (GSK) Published Kinase Inhibitor Set (PKIS) and other kinase collections have resulted in identification of hit compounds against other parasites such as Plasmodium falciparum and Trypanosoma brucei and thus provided an excellent starting point for further optimization and validation of compounds using target-based approaches (29, 31–33). Given the properties of kinases as “druggable” targets and the encouraging success of kinase inhibitors against parasitic diseases, including cryptosporidiosis, we focused our efforts on identifying new kinase inhibitors that can be repurposed as anti-cryptosporidials. Here, we report screening of 473 compounds from a GSK kinase library using a C. parvum nanoluciferase (Nluc) reporter strain that led to the identification of potent and selective compounds that inhibited parasite growth *in vitro.* These promising new inhibitors belong to the tyrosine kinase family and offer opportunities for further optimization and development as lead drug candidates against cryptosporidiosis.

## RESULTS

### GSK library screening identifies potent anti-cryptosporidial compounds.

**FIG 1:**
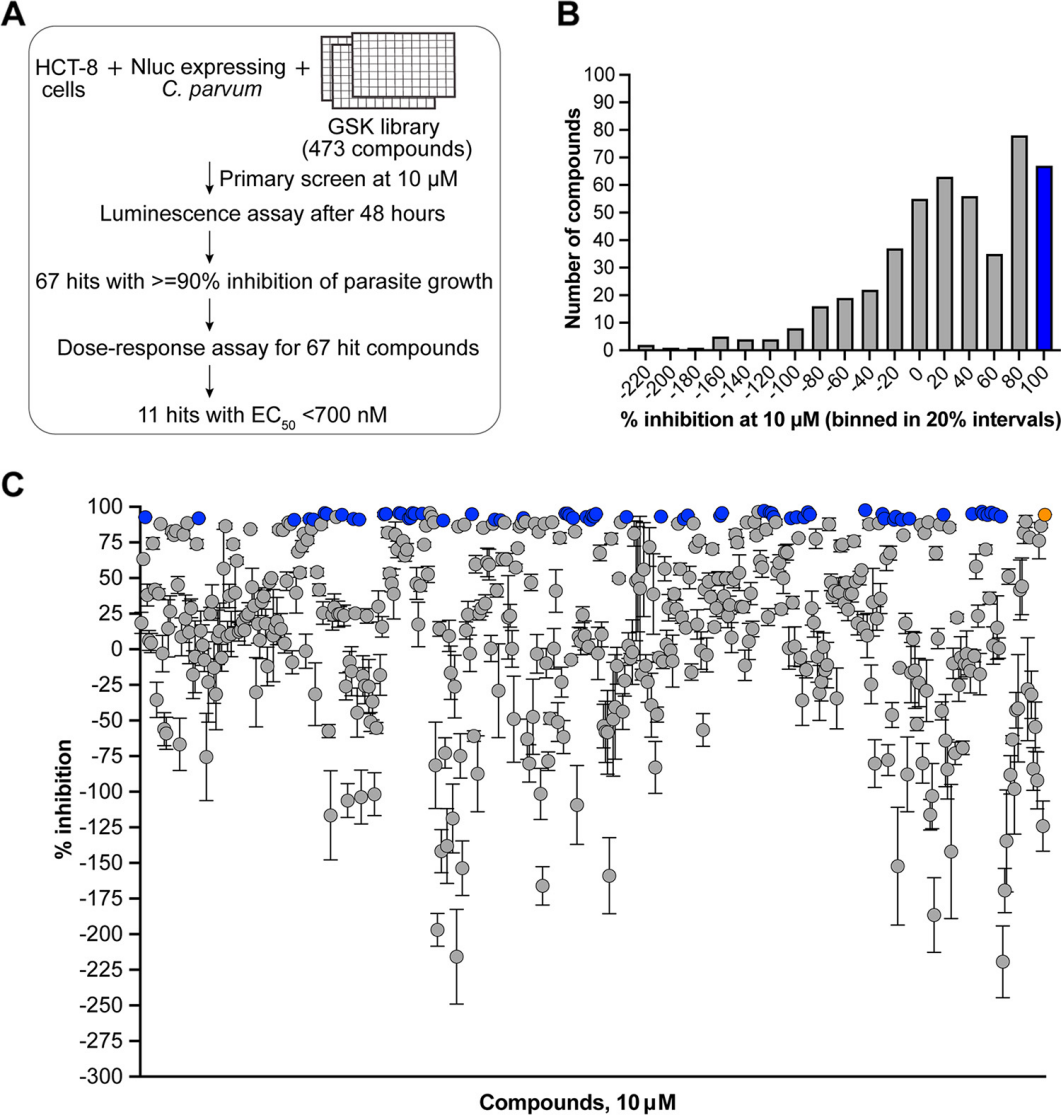
Screening of GSK kinase library against C. parvum. (A) Schematic of the screening process and identification of anti-cryptosporidial hits. (B) Histogram depicting the number of compounds and the percent inhibition at 10 μM (binned in 20% intervals). Blue bar includes the 67 compounds showing >90% inhibition during the primary screen. (C) Scatterplot of the percent inhibition of C. parvum growth for all 473 compounds. Mean and standard error of the mean (SEM) for three technical replicates are shown. Blue circles represent the 67 compounds with >90% parasite inhibition. Orange circle represents the positive control (nitazoxanide) used in the assay. Inhibition data for all 473 compounds are provided in Table S1.

To identify compounds with anti-cryptosporidial activity, we screened 473 compounds from a GSK kinase library using a robust assay (Z′> 0.6) based on transgenic C. parvum strain expressing a nanoluciferase reporter (34) (Fig. 1A, Table S1). This is an established assay for high-throughput screening of libraries and focused inhibitor collection, that has been successful in identifying new and highly efficacious anti-cryptosporidial compounds (14, 35–37). Luminescence measurements after 48 h of infection of HCT-8 cells identified compounds that inhibited C. parvum growth *in vitro*. Out of the 473 compounds tested at 10 μM in the primary screen, 67 compounds exhibited ≥90% inhibition of C. parvum growth (Fig. 1B and Fig. 1C). In all our assays, we included nitazoxanide at 10 μM as a positive control, and as expected it resulted in >90% inhibition of C. parvum growth (Fig. 1C).

### Highly efficacious compounds selectively inhibit *Cryptosporidium* compared to host cells.

**FIG 2:**
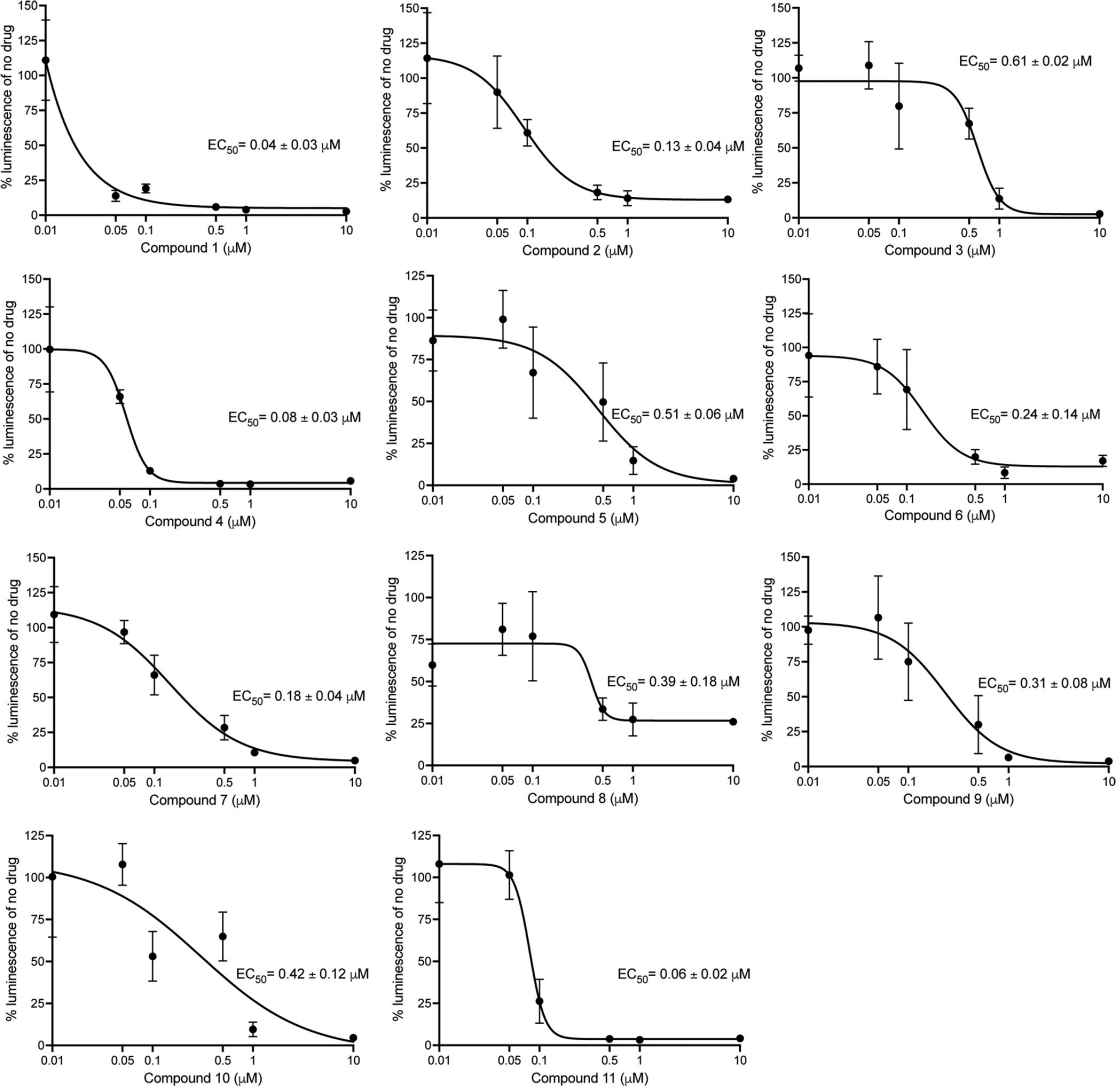
Dose-response curves and EC_50_ determination for 11 anti-cryptosporidial compounds. Mean and standard deviation (SD) of three technical replicates are shown. The experiment was repeated twice, and representative data are shown. Mean EC_50_ ± SEM values based on the two experiments are indicated on the plots.

We sought to evaluate the half-maximal effective concentration (EC_50_) of the 67 compounds identified from the primary screen in order to identify hits with nanomolar activity against C. parvum. Dose-response assays for these 67 compounds revealed 11 potent anti-cryptosporidial compounds with mean EC_50_ values ranging from < 0.04 μM to 0.61 μM (Fig. 2). In our next step, we evaluated the toxicity on host cells for those compounds that were available in enough quantity and determined their selectivity window for inhibiting C. parvum growth *in vitro*. Notably, many compounds revealed a high selectivity index (HepG2 CC_50_/C. parvum EC_50_), indicating their potential for future development (Table 1). Chemical structures of the 11 hit compounds are provided in Table 1, and in SMILES format in Table S3.

**TABLE 1:** Structures and selectivity of 11 hits against C. parvum^*a*^

Compound	Structure	*Cp* EC_50_ (μM)	HepG2 CC_50_ (μM)	Selectivity index
1	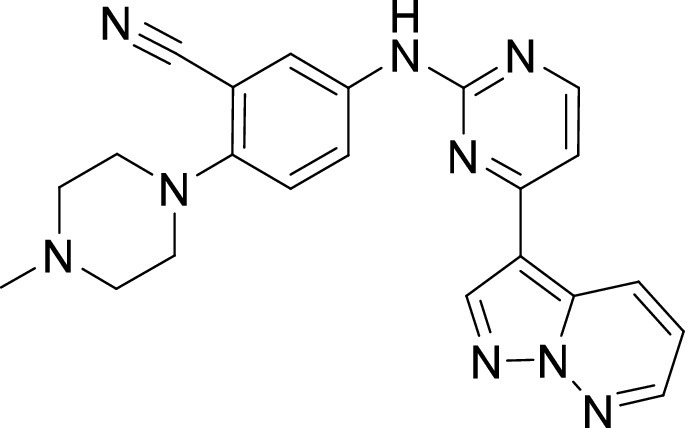	0.04	>100	>100
2	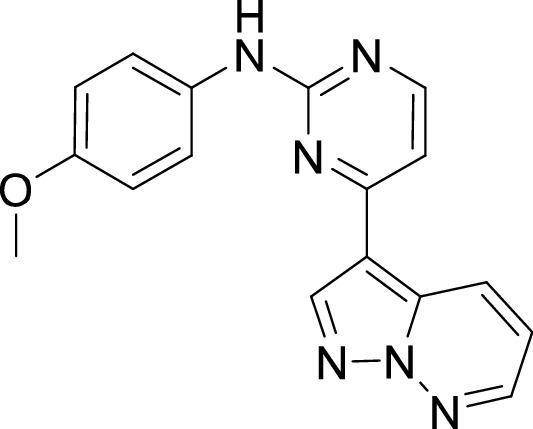	0.13	>100	>100
3	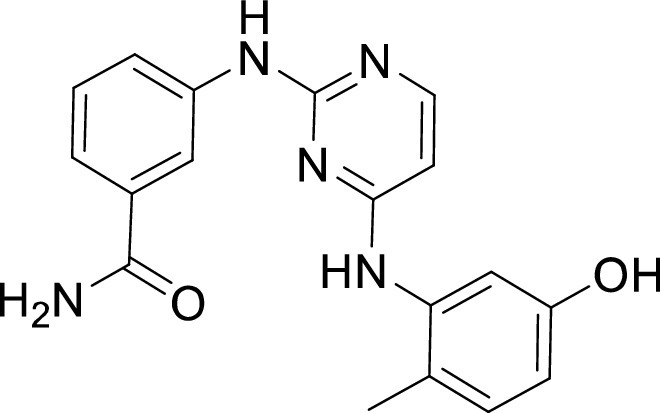	0.61	33.1	54
4	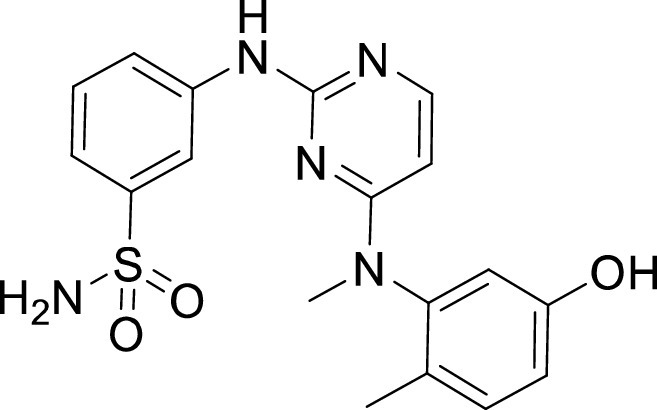	0.08	ND	ND
5	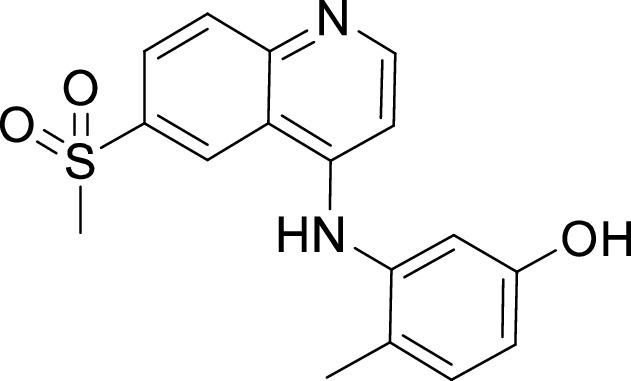	0.51	15.8	31
6	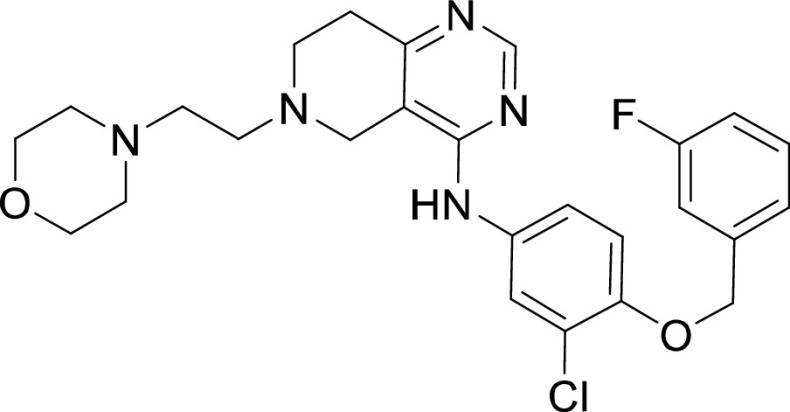	0.24	46.7	>100
7	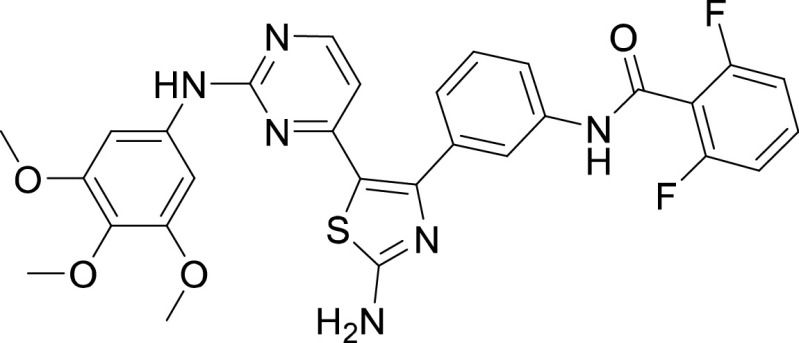	0.18	2.6	14
8	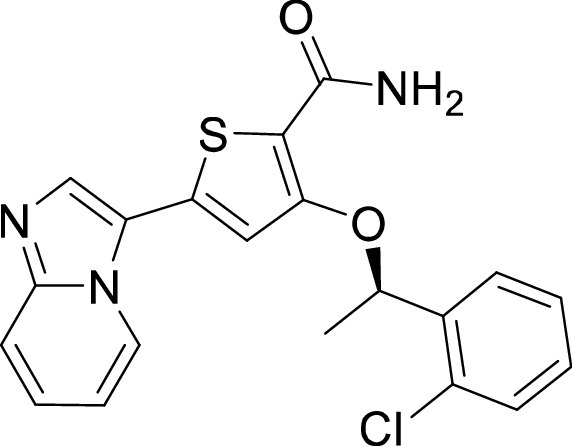	0.39	ND	ND

9	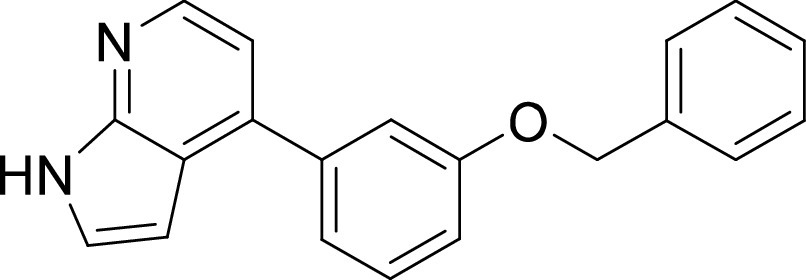	0.31	ND	ND
10	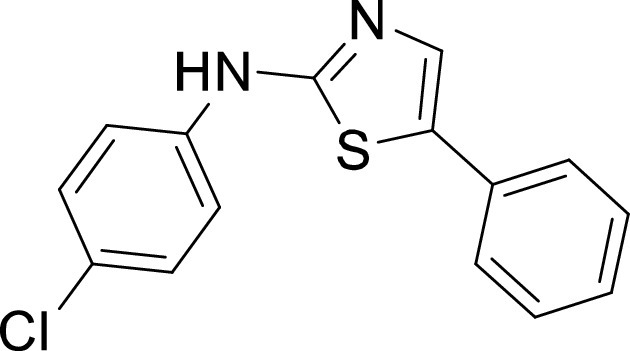	0.42	ND	ND
11	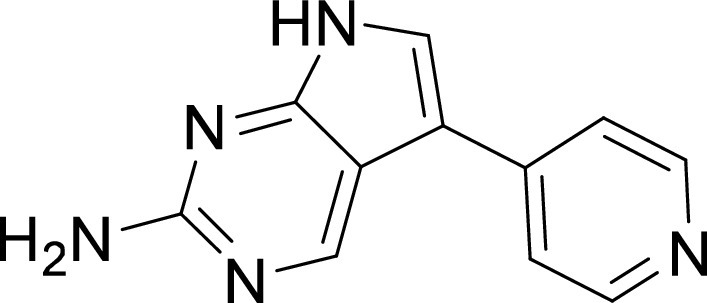	0.06	50.1	>100


a ND, not determined; due to unavailability of enough compound for the assay. Mean EC_50_ values based on two independent experiments are shown. Compound structures in SMILES format are provided in Table S3.

### Kinome profiling reveals kinases targeted by these compounds.

**FIG 3:**
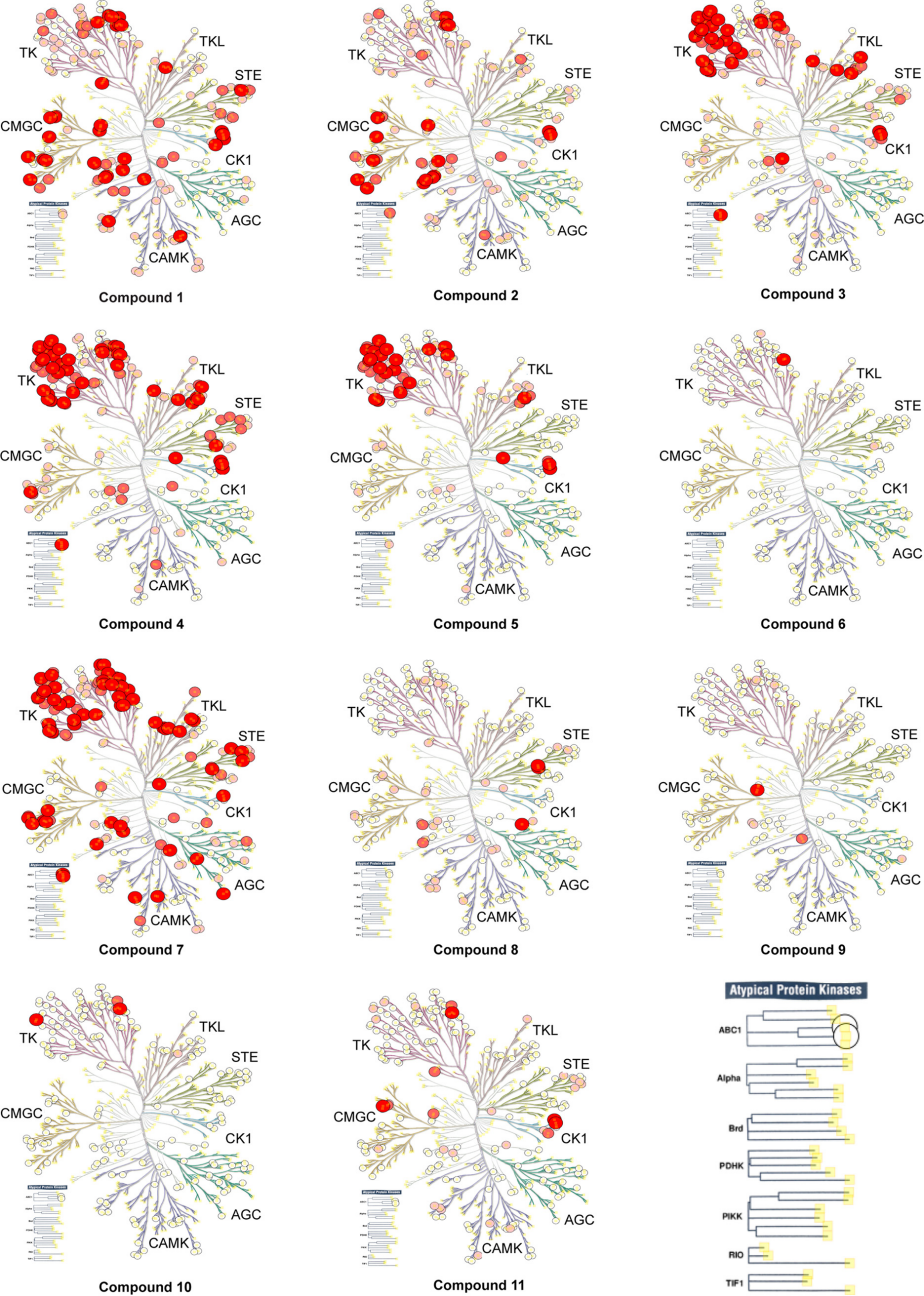
Inhibition profiles of 11 compounds across the kinome tree. The percent inhibition at 10 μM compound concentration against a panel of 203 kinases are shown. Increasing displacement of kinases from immobilized beads is represented in increasing size and color (in 4 bins with boundaries of 80%, 90.0% and 99.9%). The eight kinase groups highlighted on the tree include AGC (containing protein kinases A, G, and C families), CAMK (calcium/calmodulin-dependent protein kinase), CK1 (casein kinase 1), CMGC (containing CDK, MAPK, GSK3 and CLK families), STE (homologs of yeast sterile 7, sterile 11 and sterile 20 kinases), TK (tyrosine kinase), TKL (tyrosine kinase-like) and atypical protein kinases. Illustrations reproduced courtesy of Cell Signaling Technology, Inc. (www.cellsignal.com). The phylogram legend for atypical protein kinases, and increased font size for the eight highlighted kinases groups has been added for better visualization. The associated KINOMEscan data set is provided in Table S2.

Compounds 1 and 2 belong to a series of pyrazolopyridazines and were originally synthesized to target cyclin-dependent kinases CDK2 and CDK4 (38, 39). Consistent with the activity assays, KINOMEscan profiling revealed >99% inhibition against two CDKs (CDK2 and CDK5) and >90% against Aurora kinase A at 10 μM (Fig. 3, Table S2). Notably, a significant number of other kinases were also strongly bound (≥99%) at this concentration (50 in total for compound 1 and 28 for compound 2, not including the CDKs and Aurora kinase A), thus making it difficult to assess the degree of selectivity of these compounds for cyclin-dependent kinases over other kinase branches.

Compounds 3 and 4 belong to the bis-anilinopyrimidine class of kinase inhibitors. Compound 3 was first synthesized as an inhibitor of tyrosine kinases, including lymphocyte specific kinase (LCK), as was the des-methyl analogue of compound 4 (40). Compound 3 has been previously reported to potently bind to almost half of the tyrosine kinases tested, with subnanomolar (100 nM) K_d_ values reported for 16 kinases (41). The KINOMEscan profiling of compound 3 further confirmed that this compound is a potent inhibitor of multiple tyrosine kinases (Fig. 3, Table S2). Similar to the tyrosine kinase inhibitor compound 3, the 4-anilino quinoline compound 5 was also originally developed as an inhibitor of LCK-family tyrosine kinases (42). The activity of compound 5 was also found to be more pronounced in the LCK, SRC and Ephrin branches (Fig. 3).

Compound 6 was designed as an EGFR/ErbB2 inhibitor, with some structural similarity to Lapatinib (43). The 1-chloro-2-([3-fluorobenzyl]oxy)benzene group of Lapatinib is responsible for the strong selectivity for this family of kinases compared to other human kinases. This same group is also present in compound 6, and thus similar to Lapatinib it displayed an extremely restricted selectivity profile. Kinome profiling for compound 6 resulted in one kinase branch (EGFR) that showed >99% inhibition at 10 μM (Fig. 3, Table S2).

Compound 7 belongs to a series of 5-(pyrimidin-4-yl)thiazoles inhibitors of Erb kinases (44). Kinome profiling at 10 μM for compound 7 showed >99% inhibition against 48 kinases, including high concentration in the tyrosine kinase branch as well as activity against all kinase branches (Fig. 3, Table S2).

Compound 8 originated as a Polo-Like Kinase 1 (PLK1) inhibitor (45). Therefore, it is not surprising that this compound was found to be highly selective for PLK1 (Fig. 3). An analog from the same compound series of 3-(thiophen-2-yl)imidazo[1,2-a]pyridines has been previously reported to be highly selective for PLKs when tested against a panel of 212 kinases (45). Similar selectivity for PLKs has also been reported for the closely related compound that was tested against 196 human kinases (32).

For compounds 9, 10 and 11, we could not precisely define a particular kinase that is targeted by these compounds. A previous study had reported activity of compound 9 against JAK3 and ROCK1 (*K_i_* < 0.5 μM) (38) but kinome profiling suggested little binding of this compound to JAK kinase domains. We could not test ROCK1 since it was not represented in the commercial kinase panel used for this analysis (Fig. 3). Compound 9 showed ≥99% inhibition of two kinases (CSNK2A1 and LKB1) and ≥90% inhibition of five kinases namely, AAK1, AXL, BIKE, FLT3 and MERTK (Table S2). Compound 10 was originally described as an inhibitor of KDR (VEGFR2) and FLT1 (46). Our kinome profiling analysis indicated relatively weak inhibition of these kinases, and >99% inhibition of only three kinases (KIT, LYN and PDGFRβ) while two other kinases (FLT3 and PDGFRα) were inhibited at >90% at 10 μM (Fig. 3, Table S2). Compound 11 showed ≥99% inhibition of nine kinases that included CLK1, CLK2, CSNK1D, CSNK1E, CSNK1G1, JAK1(JH2), KIT, PDGFRα and TTK. Collectively, our data revealed compounds with anti-cryptosporidial activity that target multiple kinases, while a few were selective against particular family of kinases.

## DISCUSSION

*Cryptosporidium* is a major cause of diarrhea-associated mortality in young children; and multiple episodes of infection in surviving children have long-term consequences on growth and development (2, 4, 5). The lack of an effective drug calls for urgent development of new and effective treatments to reduce the global disease burden of cryptosporidiosis in young children and in patients with weak immune status. In this study, we screened a GSK kinase library of 473 compounds using a robust, validated assay that utilizes transgenic C. parvum expressing a nanoluciferase reporter (34) and identified 67 new compounds that inhibit parasite growth. Dose-response assays on these 67 compounds revealed 11 hits that inhibited *Cryptosporidium* growth at nanomolar concentrations. Some of these hits showed selectivity against particular kinases, while others were active across multiple kinase branches. Since these compounds originate from a human kinase library, it is possible that they may be inhibiting host kinases in addition to parasite kinases.

The pyrazolopyridazine compounds 1 and 2 showed a bias toward human CDKs but were also widely active outside that branch. Analogs from the pyrazolopyridazine series have been already released to the academic community as part of the GSK Published Kinase Inhibitor Set (PKIS) (32). These include GW779439X (PubChem CID: 10173796) which is a potent kinase inhibitor and very closely related to compound 1. Interestingly, GW779439X has also been recently reported to inhibit the bacterial penicillin binding protein and serine/threonine kinase-associated protein (PASTA) kinase Stk1 and increase the sensitivity of methicillin-resistant Staphylococcus aureus (MRSA) to *β*-lactam antibiotics (47), thus indicating the activity of this class of compounds against another serine/threonine kinase in addition to CDKs. Similarly, compounds 7, 9, 10, and 11 were not highly selective against a particular kinase and showed activity against multiple branches of kinases.

On the other hand, our results revealed five compounds that selectively inhibited specific families of kinases. These include compound 6 that was highly selective for EGFR/ErbB2 kinases, while compound 8 was a selective PLK inhibitor. A set of particularly interesting and potent inhibitors that have emerged from our analysis are compounds 3, 4, and 5. These three compounds exhibited selective targeting of tyrosine kinases, and two of these compounds show a safety window for future development given their low cytotoxicity to host cells. The selective targeting of these kinases is intriguing since *Cryptosporidium* does not express any tyrosine kinases. However, its genome encodes three tyrosine kinase-like enzymes (TKLs) (48). TKLs in other protozoan parasites exhibit sequence similarity to tyrosine kinases but function as serine-threonine kinases (49, 50). TKLs have also emerged as attractive drug targets in other parasites. For example, human tyrosine kinases inhibitors have also been reported to be potent against Trypanosoma brucei and *Plasmodium* s*pp*, although like *Cryptosporidium* these parasites also do not express canonical tyrosine kinases (51–54). Therefore, it is possible that all three compounds (compounds 3, 4, and 5) inhibit one or more TKLs in C. parvum. Future studies on target validation of these TKLs in C. parvum by gene deletion or conditional knockout approaches will allow determination of their functional role in parasite biology. Based on their essentiality, target-based drug discovery approaches could then be applied against one particular C. parvum TKL, or multiple TKLs could be targeted in a polypharmacology strategy. Lead optimization of TKL inhibitors would require improving selectivity against the parasite, reducing off-target kinase activity and characterizing pharmacokinetic properties. Lead compounds can then be further evaluated for their efficacy in killing *Cryptosporidium* using immunocompromised mice and clinical calf models for cryptosporidiosis. During the development process, it is important to consider gastrointestinal exposure of compounds due to the peculiar epicellular attachment mechanism of *Cryptosporidium* to the intestinal epithelial cell that involves its encapsulation by host villus membrane and membranous structures that separate parasite from host cytoplasm. For BKIs targeted against C. parvum CDPK1, a high drug exposure in the intestine rather than systemic exposure has been reported to be a better predictor of *in vivo* efficacy (55). Similarly, KDU731 compound targeted against C. parvum PI(4)K, displayed moderate to-low bioavailability that correlated with its efficacy in animal models of infection (14).

Interestingly, in addition to compounds that inhibited growth of C. parvum, our phenotypic screen also identified several compounds that enhanced parasite growth (Fig. 1C, Table S1). We speculate that host kinases may be involved in preventing *Cryptosporidium* from establishing infection, and inhibition of these kinases could possibly have promoted parasite infection and replication. Although *Cryptosporidium* has been reported to manipulate host signaling machinery by activating kinases for actin polymerization and cell invasion (56, 57) the function of host kinases and/or their interaction with parasite effectors in restricting infection are not known.

Overall, our study identified new, potent and selective compounds from a human kinase library that can effectively block C. parvum growth. The discovery of these promising new kinase inhibitors provides opportunities for their further optimization and development as lead anti-cryptosporidial agents.

## MATERIALS AND METHODS

### Animal experiments.

All mice procedures reported in this study were approved by the Institutional Animal Care and Use Committee (IACUC) of the University of Illinois at Urbana-Champaign under protocol numbers 17188 and 20188. Breeder pairs of interferon gamma knock out (IFN-γ KO) mice (B6.129S7-*Ifng^tm1Ts^*/J) were purchased from the Jackson laboratory for mating, and an in-house mouse colony was maintained. Four- to 6- week old IFN-γ KO mice (*n* = 5 mice per cage) were used for generating and passaging stable transgenic C. parvum strain. Both male and female IFN-γ KO mice (age- and sex-matched) were used for infection experiments. Mice infected with C. parvum were monitored for weight loss, fur ruffling, hunched posture and inactivity, and any animal showing a weight loss of ≥= 15% was euthanized.

### Kinase library.

An in-house library of 473 human kinase inhibitors was obtained from GSK, Tres Cantos. These were the available samples remaining from a set of 577 compounds selected to represent the diversity of the GSK kinase collection (41). The compounds were provided as a 10 mM stock in dimethyl sulfoxide (DMSO) on 96-well plates and stored at −80°C until use in assays.

### Cryptosporidium parvum strain expressing nanoluciferase (Nluc).

Cryptosporidium parvum oocysts (AUCP-1 strain, kind gift from Mark Kuhlenschmidt’s laboratory, University of Illinois at Urbana-Champaign) were used to generate a transgenic strain that expresses the Nluc reporter gene. To generate this strain, the C. parvum thymidine kinase (*tk*) gene was replaced by a cassette consisting of Nluc fused to the Neomycin-resistance marker (Nluc-Neo) using CRISPR/Cas9 gene editing and IFN-γ KO mouse infection model using the protocols described previously (34). Feces collected from mice subjected to oocyst purification using sucrose flotation and cesium chloride density gradient centrifugation. Purified oocysts were used to further passage this strain into new cages of IFN-γ KO mice for generating additional oocysts for use in *in vitro* drug assays.

### *In vitro* C. parvum growth inhibition assay.

Host intestinal epithelial adenocarcinoma (HCT-8) cells were seeded into 96-well plates and grown to 70% confluence in RPMI medium supplemented with l-glutamine, 10% fetal bovine serum (FBS), 0.1 U/mL penicillin, 0.1 μg/mL streptomycin, 0.25 μg/mL amphotericin B and 1 mM sodium pyruvate. The host cell medium was aspirated and replaced with *Cryptosporidium* infection media (RPMI containing 2% FBS, 0.1 U/mL penicillin, 0.1 μg/mL streptomycin and 0.25 μg/mL amphotericin B) media prior to infection. The HCT-8 cells were infected with bleached and washed Nluc expressing C. parvum oocysts (2,000 oocysts per well) to which 10 μM compounds were added and plates were incubated for 48 h at 37°C, 5% CO_2_ and luminescence was measured using the protocol described previously (14, 58). All 473 compounds were tested in triplicate wells. Negative (no drug) and positive control (10 μM nitazoxanide) wells (three replicates each) were also set-up in the same plate. After the incubation, medium was removed and 100 μL of NanoGlo lysis buffer (Promega) was added to the wells followed by incubation for 15 min at 37°C, 5% CO_2_. The lysate was suspended by pipetting and 100 μL of NanoGlo lysis buffer containing 1:50 of NanoGlo substrate (Promega) was added to each well. The entire lysate was transferred to white 96-well plate and luminescence was measured on the Wallac VICTOR2 1420 multilabel counter (Perkin Elmer Inc.). Compounds showing > 90% inhibition compared with no drug control were subjected to a dose response assay (10 nM to 10 μM compound) to determine the half-maximal effective concentration (EC_50_). For the dose response assay, compounds were tested in triplicate wells in two independent experiments and percent luminescence of no drug was calculated.

### Host cell cytotoxicity assays.

Host cell cytotoxicity of selected top and available compounds (up to 100 μM concentration) were performed on HepG2 cells at GSK. HepG2 cells were grown in Eagle’s Minimum Essential Medium (MEM) containing 10% FBS, 1% nonessential amino acids (NEAA) and 1% penicillin/streptomycin to >50% confluence prior to harvesting. Cells were suspended in media and 3000 cells per well were added onto 384-well clear bottom plates. Prior to addition of the cell suspension, the screening compounds (250 nl) were predispensed into plates using a liquid handler. Plates were incubated for 48 h at 37°C, 5% CO_2_. After 48 h of incubation, plates were equilibrated at room temperature for 30 min before luminescence signal was measured using CellTiter-Glo Reagent. Plates were kept at room temperature for 10 min to stabilize the signal and then read using a ViewLux luminescence reader. The HepG2 CC_50_ (from the host cell cytotoxicity assays) and C. parvum mean EC_50_ (from parasite growth inhibition assays) values were used to calculate selectivity index.

### Kinase interaction map.

All compounds were screened at DiscoverX, initially *n* = 1 at 10 μM compound concentration against a panel of 203 kinases using KINOMEscan phage display technology (41, 59). The kinases were expressed as fusions to T7 bacteriophage. This is a sensitive, competitive binding assay where test compounds were used to displace immobilized ATP-site binding probe ligands from kinases of interest. The amount of kinase that remained bound to the immobilized probe ligands on the solid support was quantified. Percentage inhibition data were was plotted on kinome trees using the web-based tool KinMap (http://www.kinhub.org/kinmap) (60).

### Statistical analysis.

EC_50_ values were calculated using a nonlinear regression (curve fit of log-dose versus response) in GraphPad Prism version 8.
