# Supplementary material for: Tyrosine Kinase Inhibitors Display Potent Activity against Cryptosporidium parvum
Source: Microbiol Spectr. 2022 Dec 19;11(1):e03874-22. doi: 10.1128/spectrum.03874-22 (PMC9927415; doi:10.1128/spectrum.03874-22)
Supplement: Supplemental file 4 — Legends of Tables S1 to S3. Download spectrum.03874-22-s0004.pdf, PDF file, 0.04 MB [file spectrum.03874-22-s0004.pdf]

Supplementary table legends

Table S1: Mean percent inhibition data for all 473 compounds. The 67 hit compounds are highlighted in blue text.

Table S2: KINOMEscan dataset for compounds 1-11. Percent inhibition (all n=1, 10 μM).

Table S3: Chemical structure of compounds in SMILES (Simplified molecular-input line-entry system) format.
